# *N*-Acyl Homoserine Lactone Production by *Klebsiella pneumoniae* Isolated from Human Tongue Surface

**DOI:** 10.3390/s120303472

**Published:** 2012-03-12

**Authors:** Wai-Fong Yin, Kathiravan Purmal, Shenyang Chin, Xin-Yue Chan, Chong-Lek Koh, Choon-Kook Sam, Kok-Gan Chan

**Affiliations:** 1 Division of Genetics and Molecular Biology, Institute of Biological Sciences, Faculty of Science, University of Malaya, 50603 Kuala Lumpur, Malaysia; E-Mails: yinwaifong@yahoo.com (W.-F.Y.); shenyang86@yahoo.com (S.C.); evepy88@yahoo.com (X.-Y.C.); 2 School of Dental Sciences, Universiti Sains Malaysia, 16150 Kubang Kerian, Kelantan, Malaysia; E-Mail: drkathi@um.edu.my; 3 Natural Sciences and Science Education AG, National Institute of Education, Nanyang Technological University, 1 Nanyang Walk, 637616, Singapore; E-Mails: chonglek.koh@nie.edu.sg (C.-L.K.); choonkook.sam@nie.edu.sg (C.-K.S.)

**Keywords:** *N*-acylhomoserine lactone, *N*-3-dodecanoyl-l-homoserine lactone (C12-HSL), *N*-heptanoyl-l-homoserine lactone (C7-HSL), *N*-octanoyl-l-homoserine lactone (C8-HSL), oral cavity, posterior dorsal surface, quorum sensing, tongue

## Abstract

Bacteria communicate by producing quorum sensing molecules called autoinducers, which include autoinducer-1, an *N*-hexanoyl homoserine lactone (AHL), and autoinducer-2. Bacteria present in the human oral cavity have been shown to produce autoinducer-2, but not AHL. Here, we report the isolation of two AHL-producing *Klebsiella pneumoniae* strains from the posterior dorsal surface of the tongue of a healthy individual. Spent culture supernatant extracts from *K. pneumoniae* activated the biosensors *Agrobacterium tumefaciens* NTL4(pZLR4) and *Escherichia coli* [pSB401], suggesting the presence of both long and short chain AHLs. High resolution mass spectrometry analyses of these extracts confirmed that both *K. pneumoniae* isolates produced *N*-octanoylhomoserine lactone and *N*-3-dodecanoyl-l-homoserine lactone. To the best of our knowledge, this is the first report of the isolation of *K. pneumoniae* from the posterior dorsal surface of the human tongue and the production of these AHLs by this bacterium.

## 1. Introduction

The human oral cavity contains more than 700 species of microorganisms [1]. This plethora of bacteria lives in harmony in a healthy individual, therefore it is logical to visualise a dynamic interaction between the host environment and interaction among the oral bacterial consortium. While streptococci represent the major genus, other bacteria, including *Actinomyces* spp., *Capnocytophaga* spp., *Eikenella* spp., *Haemophilus* spp., *Prevotella* spp., *Propionibacterium* spp., *Veillonella* spp. and *Fusobacterium* spp., are commonly found in the oral cavity [2]. Hence the oral cavity provides a unique environment for microbial interaction and communication. Most oral bacteria, which include *Prevotella intermedia*, *Porphyromonas gingivalis*, *Streptococcus gordonii* and *Streptococcus mutans*, produce autoinducer-2 (AI-2) [3–7].

**Figure 1.**
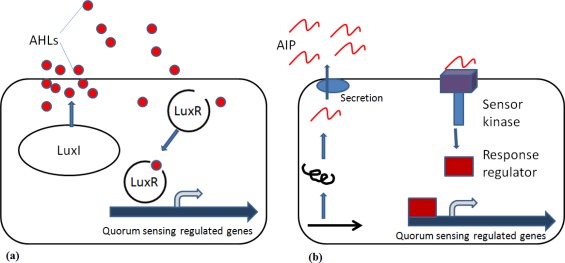
Schematic presentations of bacterial quorum sensing systems. (**a**) In Gram-negative bacteria, AHLs (filled circles) are produced by the LuxI synthase and will bind to the cognate LuxR receptor. The AHL-LuxR protein complex will bind to promoter DNA elements and regulate transcription of QS-regulated genes. (**b**) Gram-positive bacteria synthesize AIP (curvy lines) that are post-translationally modified and secreted. AIP detection occurs via a two-component signal transduction circuit, leading to the ATP-driven phosphorylation of a response regulator protein, which then binds to promoter DNA and regulates transcription of QS-regulated genes.

Gram-negative and Gram-positive bacteria communicate by a process termed quorum sensing (QS), which typically involves the production and sensing of QS molecules called autoinducers to link the surrounding cell density with activation of appropriate compensatory regulation of cell function (Figure 1). In Gram-negative bacteria, *N*-acylhomoserine lactones (AHLs) are arguably the most studied QS signalling molecules. AHLs are usually produced by a protein homologous to LuxI and they will in turn bind to LuxR protein [8]. Once the threshold level of an AHL is detected, transduction leads to the induction of genes that control a variety of survival functions, including the production of antimicrobial substances and protection against the host’s defence mechanisms [9,10].

In Gram-positive bacteria the signalling molecules are post-translationally modified peptides, known as autoinducer peptides (AIP) (Figure 1). Thus far, the only QS mechanism shared by both Gram-positive and Gram-negative bacteria involves the production of AI-2 by the enzyme LuxS. AI-2 appears to be an important signal molecule among different species in a mixed-species community [11] and it differs from AHL, which regulates gene expression in genetically identical cells.

Both Gram-positive and Gram-negative bacteria have been implicated in several systemic infections like bacterial endocarditis, aspiration pneumonia, osteomyelitis in children, preterm low birth weight, cardiovascular disease and dental diseases like caries, periodontal disease and dentoalveolar abscess [1]. Recently, we have analysed the pathogenic *Aeromonas* sp. from various parts of the human body and found that AHL production is very common in this bacterium [12]. Hence, it is of clinical importance to study the role of autoinducers produced by oral pathogens in determining the health of an individual. Hitherto, there has been no report of AHL production by oral bacteria [2,6,13]. Here we report the isolation of two strains of AHL-producing *Klebsiella pneumoniae* from the tongue surface of a healthy individual and the characterization of the AHLs produced.

## 2. Experimental Section

### 2.1. Bacterial Strains

Besides the oral *K. pneumoniae* isolated in this work, *Escherichia coli* DH5α was used as a host for DNA manipulations. Bioreporters *E. coli* [pSB401] [14] and *Agrobacterium tumefaciens* NTL4(pZLR4) [15] were used. In the *lux*-based plasmid-bearing AHL biosensor *E. coli* [pSB401], the *Photorhabdus luminescens luxCDABE* cassette has been fused to the *Vibrio fischeri luxI* promoter and contains the *V. fischeri luxR* gene [14]. Activation of *LuxR* by exogenous *N*-3-oxohexanoyl-l-homoserine lactone (3-oxo-C6-HSL) or a related AHL leads to the expression of *luxCDABE* and the emission of light. This biosensor is versatile and able to report the structural differences inherent in the AHL family since it responds differentially to AHL molecules with variable acyl side chain lengths. *A. tumefaciens* NTL4(pZLR4) is an NT1 derivative carrying a *traG::lacZ* reporter fusion. This strain does not produce its own AHLs, but the *traG::lacZ* reporter gene is induced only when its transcription activator TraR detects a cognate exogenous AHL. Induction of the reporter gene, leading to production of β-galactosidase enzyme, is measured by using X-gal, a β-galactosidase substrate, for colorimetric (blue pigmentation) detection. *A. tumefaciens* NTL4(pZLR4) was cultured in AB medium or agar (solidified with bacto-agar at 15 g/L), supplemented with gentamicin (150 μg/mL) and glucose (0.5%, w/v) [15]. For AHL detection with *A. tumefaciens* NTL4(pZLR4), AB agar was supplemented with X-gal (60 μg/mL, final concentration). All other bacteria were routinely cultured in Lysogeny Broth (LB) medium (in grams per litre: tryptone, 10; yeast extract, 5; and NaCl, 5), without or with bacto-agar (15 g/L), buffered with 50 mM 3-[*N-*morpholino] propanesulfonic acid (MOPS) to pH 5.5. When necessary, growth media were supplemented with ampicillin (100 μg/mL). *A. tumefaciens* NTL4(pZLR4) was grown at 28 °C, whereas *E. coli* and oral bacteria were grown at 37 °C.

### 2.2. Enrichment of Bacteria from Tongue Surface Debris

This study was approved by the Ethics Committee of the Faculty of Dentistry, University of Malaya. Tongue surface debris was collected from an individual with healthy oral condition in 2008 at the Faculty of Dentistry, University of Malaya. A sterile stainless steel tongue scraper was used to gently scrape the tongue surface to collect the specimen. The debris on the scraper was suspended in 300 μL of sterile saline. To isolate bacteria from the tongue debris, we first evaporated 5 μL of *N*-heptanoyl-l-homoserine lactone (C7-HSL, 0.1 M), dissolved in absolute ethanol, to dryness and then added the 300 μL of tongue debris to rehydrate the dried C7-HSL. After mixing, 1 μL of NH_4_Cl (0.3 g/L) was added and the total volume was brought to 3 mL with KG medium [16]. The mixture was incubated at 37 °C (220 rpm) for 48 hours and transferred to fresh NH_4_Cl and C7-HSL supplemented KG medium at 48 hours interval. Aliquots were withdrawn at the fourth enrichment and spread on LB agar. Pure colonies were obtained by repeated streaking on LB agar. We used C7-HSL, rather than the commonly used even-numbered AHLs [16,17], in this study because we wished to check if any novel bacteria from the tongue surface could be isolated using C7-HSL as the sole carbon source.

### 2.3. Strain Identification

All DNA manipulations and PCR amplification of 16S rDNA genes with the universal primer pairs 27F and 1525R were carried out as described [18,19]. T7 and SP6 universal primers and internal primers previously designed to anneal to internal target regions of the 16S rDNA were used [20]. Nucleotide sequence alignments and phylogenetic analysis were carried out using LASERGENE software package (DNASTAR, Inc.) and MEGA software version 4.0 [21], respectively. Trees were generated using the Neighbour-Joining algorithm on aligned 16S rDNA gene sequences. Bootstrap analyses for 1,000 resamplings were used to ensure the robustness and reliability of trees constructed.

### 2.4. Extraction of AHLs from Spent Bacterial Culture Supernatants

An overnight bacterial culture was inoculated into 100 mL of LB broth. Cells were grown to OD_600_ of 1.0 and the spent culture supernatant was extracted twice with an equal volume of ethyl acetate. The organic layer was collected in a separation funnel, dried over anhydrous magnesium sulphate, filtered, and evaporated to dryness. Residues were dissolved in 100 μL of acetonitrile and stored at −20 °C. AHL extracts were analysed with a luminometer–spectrophotometer used in conjunction with *lux*-based biosensor *E. coli* [pSB401], thin layer chromatography and liquid chromatography mass spectrometry.

### 2.5. Separation and Detection of AHLs by Thin Layer Chromatography (TLC) Analysis

TLC was performed according to the protocol of Shaw *et al*. [22]. A TLC tank was filled with methanol (60%, v/v) and left for 1 hour to enable saturation. The ethyl acetate extracts (20 μL) and AHL standards (dissolved in acetonitrile) were chromatographed on an RP-18 F254s aluminium plate (20 × 20 cm; Merck, Germany). The AHL standards used were 3-oxo-C6-HSL (0.0008 μg per lane), *N*-hexanoyl-l-homoserine lactone (C6-HSL) (0.01 μg per lane), *N*-octanoyl-l-homoserine lactone (C8-HSL) (0.18 μg per lane), and *N*-decanoyl-l-homoserine lactone (C10-HSL) (0.3 μg per lane), all obtained from Sigma-Aldrich. After chromatography, the plate was air-dried and overlaid with a thin film of X-gal-containing AB agar seeded with overnight-grown *A. tumefaciens* NTL4(pZLR4). The TLC plate was then incubated for 24 to 48 hours at 28 °C. AHLs were detected by blue pigmentation on the TLC plate, and the result was digitally recorded. Duplicate experiments were carried out.

### 2.6. Measurement of Bioluminescence

To measure bioluminescence, an overnight culture of *E. coli* [pSB401] was diluted with LB to OD_600_ 0.01, and 200-μL aliquots of the diluted cells were added to each well of a 96-well optical bottom microtitre plate. AHL extracts from oral bacteria, AHL solvent (ethyl acetate) (control), and *N*-butanoyl-l-homoserine lactone (C4-HSL) (100 mM, Sigma-Aldrich) (positive control) were added to the wells containing *E. coli* [pSB401] cells, and incubated at 37 °C for 24 hours in a luminometer-spectrophotometer. Bioluminescence was measured as a function of cell density at 30 minutes interval using a combined automated luminometer–spectrophotometer (Infinite, Tecan). Growth measurement and bioluminescence were the means of triplicate experiments. Data were presented as graph RLU (Relative Light Units)/OD_495 nm_ against time, indicating approximate light output per cell.

### 2.7. Mass Spectrometry (MS) Analyses of AHLs

High resolution MS was performed as described [23] using the Agilent RRLC 1200 system coupled with an Agilent ZORBAX Rapid Resolution HT column (100 mm × 2.1 mm, 1.8 μm particle size), carried out at 60 °C, flow rate 0.3 mL/min, with injection volume 20 μL. Mobile phases A and B were 0.1% v/v formic acid in water and 0.1% v/v formic acid in acetonitrile, respectively, and the gradient profile is given in Table 1.

The high resolution ESI-MS and ESI-MS/MS analyses were performed on an Agilent 6500 Q-TOF system, operated in the ESI-positive mode, with probe capillary voltage set at 3,000 V; desolvation temperature 350 °C; sheath gas 11 mL/h; and nebulizer pressure 50 psi. Nitrogen gas was used as the collision gas in the collisionally induced dissociation mode for the MS/MS analysis, with collision energy set at 20 eV. Agilent MassHunter software was used to analyze the MS data.

## 3. Results and Discussion

### 3.1. Characterization of Oral K. pneumoniae

**Figure 2.**
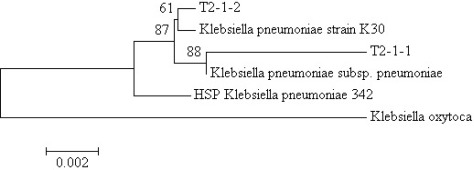
16S rDNA-based phylogenetic tree showing the phylogenetic positions of oral bacterial isolates T2-1-1 and T2-1-2. A total of 912 unambiguously aligned nucleotides were analysed using *MEGA* 4. The percentages of replicate trees in which the associated taxa clustered together in the bootstrap test are shown next to the branches. The tree is drawn to scale, with branch lengths in the same units as those of the evolutionary distances used to infer the phylogenetic tree. The bar at the bottom represents evolutionary distance as 0.002 change per nucleotide position. GenBank accession numbers are given in parentheses: *K. oxytoca* (AB476819), *K. pneumoniae* subsp. *pneumoniae* (CP000647), *K. pneumoniae* strain K30 (EU661377), and *K. pneumoniae* 342 (CP000964).

The growth medium became turbid within 48 hours after inoculation with the tongue surface debris suggesting bacterial growth. At the end of the enrichment process, cell suspensions were serially diluted and streaked on LB agar. Pure colonies with distinctive morphologies were obtained after several successive streaks. Isolates T2-1-1 and T2-1-2 from the tongue surface debris specimen were selected for further analysis. The partial 16S rDNA sequences of T2-1-1 and T2-1-2, each comprising 1,532 nucleotides, were deposited in the GenBank under accession numbers HQ907955 (T2-1-1) and HQ907956 (T2-1-2). Web-based search and phylogenetic analysis showed that both T2-1-1 and T2-1-2 were *K. pneumoniae* (Figure 2).

### 3.2. Detection of AHLs

**Figure 3.**
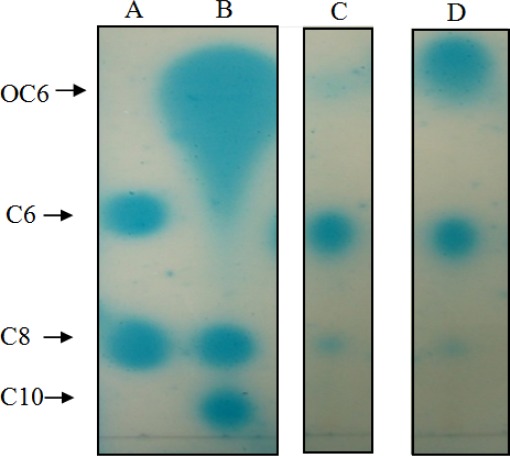
TLC analysis of AHL production by oral *K. pneumoniae* strains. Arrows indicate the positions of AHL standards run on the same plate. Lane A: C6-HSL (C6) and C8-HSL (C8). Lane B: 3-oxo-C6-HSL (OC6), C8-HSL (C8), and C10-HSL (C10). Lanes C and D: Extracts of spent culture supernatants from T2-1-1 and T2-1-2, respectively.

We succeeded in our initial objective of isolating quorum quenching bacteria from the human tongue surface (data not shown). We then tested these isolates for AHL production. To our surprise, isolates T2-1-1 and T2-1-2 produced AHLs, detected initially with *A. tumefaciens* NTL4(pZLR4) on agar plates–(data not shown). Subsequently, TLC results of the extracts of spent culture supernatants from T2-1-1 and T2-1-2 revealed three well-resolved spots with relative migration factor (*R_f_*) values corresponding to those of 3-oxo-C6-HSL, C6-HSL, and C8-HSL (Figure 3). Strain T2-1-2 appeared to produce more 3-oxo-C6-HSL than T2-1-1.

**Figure 4.**
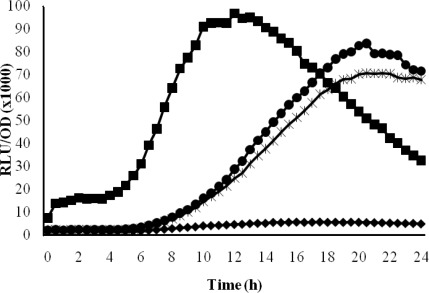
Measurement of bioluminescence. Bacterial bioluminescence and OD were measured throughout growth for 24 hours at 37 °C in the presence of synthetic C4-HSL (square) (positive control), ethyl acetate (diamond) (negative control), and AHL extracts from T2-1-1 (*K. pneumoniae*) (asterisk) and T2-1-2 (*K. pneumoniae*) (circle). *E. coli* [pSB401] served as the biosensor. Data represent means from three replicates.

The production of AHLs by T2-1-1 and T2-1-2 was confirmed by the activation of bioluminescence of *E. coli* [pSB401] (Figure 4). However, the light emission was delayed in the presence of AHL extracts from strains T2-1-1 and T2-1-2 as compared to the positive control (Figure 4). This is because in the positive control, C4-HSL was added exogenously in a sufficient amount (100 mM) that triggered bioluminescence earlier.

### 3.3. MS Analyses of the Extracts of Spent Culture Supernatants from Oral Bacteria

**Figure 5.**
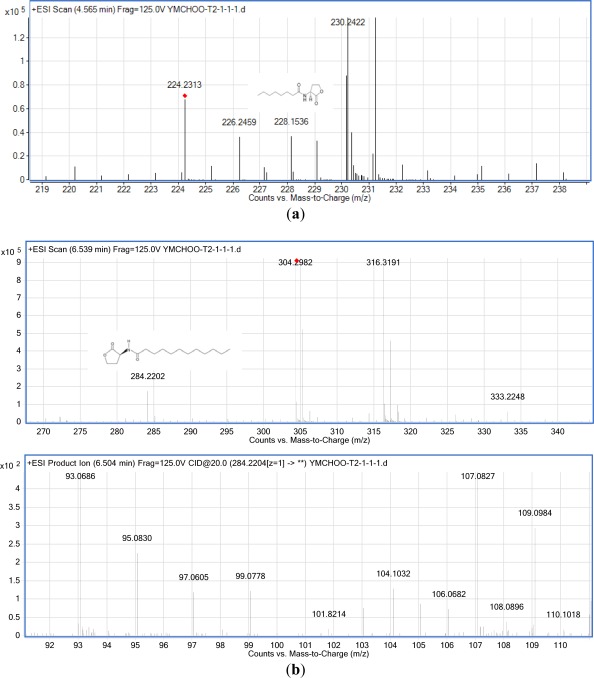
MS analyses of the extract of spent culture supernatant from oral strain *K. pneumoniae* (T2-1-1). (**a**) Mass spectra of C8-HSL (*m/z* 228.1536). (**b**) Mass spectra of C12-HSL: the upper panel shows the parent ion (*m/z* 284.2202) while the lower panel shows the product ions of C12-HSL (*m/z* 95.0830, 109.0984).

The results of MS (Figure 5) confirmed the presence of C8-HSL (*m/z* 228.1536) and C12-HSL (*m/z* 284.2202) in the spent culture supernatant from *K. pneumoniae* (T2-1-1). The ESI-MS/MS spectrum of C12-HSL shows fragments (*m/z* 95.0830, 109.0984) typical of lactone-moiety [24]. A similar approach confirmed that *K. pneumoniae* (T2-1-2) also produced C8-HSL and C12-HSL (the mass spectra for T2-1-2 are not shown). Table 2 summarizes the MS analyses of these AHLs.

Although TLC analysis indicated the presence of C6-HSL and 3-oxo-C6-HSL in the spent culture supernatants from T2-1-1 and T2-1-2, MS did not detect them. The failure to detect short chain AHLs may be due to their instability during MS analysis in our experimental condition or their degradation by T2-1-1 and T2-1-2 during extraction.

Another possible reason is that AHL biosensors, especially *A. tumefaciens* NTL4(pZLR4), are less reliable although the biosensor-TLC assay method provides a quick and convenient way to detect AHLs, providing the basis for high sensitivity detection by LC-MS/MS and nuclear magnetic resonance spectroscopy. Hence, in this study, we used the high resolution MS analysis, which provides high accuracy of mass determination, to confirm the presence of these AHLs in spent culture supernatants from *K. pneumoniae*.

*K. pneumoniae* and *K. oxytoca* strains have been reported to produce high AHL activities only when grown microaerophilically in LB medium, but insignificant amounts of AHLs when grown in LB medium under aerobic condition [25]. In contrast, in this study *K. pneumoniae* was shown to produce detectable amounts of AHLs when grown aerobically in LB medium buffered with MOPS. Because AHLs have short half-life in alkali condition [26], the MOPS-buffered LB medium provides protection to AHLs against lactonolysis at basic pH, hence stabilising AHLs and enabling them to be readily detected in our study.

Although AHLs have well defined immune modulatory effects *in vitro* and are detectable in body fluids (such as sputum from cystic fibrosis patients infected with *Pseudomonas aeruginosa*) [27], the long term effects of ingesting AHLs into the digestive tracts of human beings remain unknown. We had performed an acid tolerance test on T2-1-1 and T2-1-2, and both isolates were viable when grown for 24 hours in LB buffered at pH 5. However, they did not remain viable when grown in pH > 3 (data not shown). These results led us to speculate that when these AHL-producing bacteria are dislodged from the tongue surface, they may survive gastric juice and persist in human bowels. During the passage from the oral cavity through the digestive tracts, these strains may produce AHLs that could have an impact on the host *in vivo*.

In this study, *K. pneumoniae*, which is not commonly found in the oral cavity, was isolated from the posterior dorsal surface of the human tongue. To the best of our knowledge, this is the first report of AHL-producing bacteria on the human tongue surface. Hitherto, oral bacteria have only been reported to produce AI-2, synthesized by LuxS [2,3,5,6]. This study has established the production of AHLs by oral bacteria and has widened the scope of QS research in oral cavity from AI-2 to AHL.

## 4. Conclusions and Outlook

The tongue surface is a rich source of bacteria including QS bacteria that rely on AHLs as signalling molecules. Here, oral *K. pneumoniae* isolated from the human tongue surface has been confirmed to produce AHLs by biosensors and high resolution MS.

## Figures and Tables

**Table 1.** Liquid chromatography gradient.

**Time (min)**	**Mobile phase A (0.1% v/v formic acid in water)**	**Mobile phase B (0.1% v/v formic acid in acetonitrile)**

0.0	60%	40%
5.0	20%	80%
7.0	5%	95%
10.0	5%	95%
11.0	60%	40%
13.0	60%	40%

**Table 2.** MS analyses of AHLs in the extracts of spent culture supernatants from oral *K. pneumoniae*.

**Bacterial strain**	**Mass spectra**

*K. pneumoniae* (T2-1-1)	C8-HSL (*m/z* 228.1536)
	C12-HSL (*m/z* 284.2202; MS/MS: *m/z* 95.0830, 109.0984)
*K. pneumoniae* (T2-1-2), mass spectra not shown	C8-HSL (*m/z* 228.1541)
	C12-HSL (*m/z* 284.2202; MS/MS: *m/z* 95.0828, 109.0977)

## References

[1] Aas J.A., Paster B.J., Stokes L.N., Olsen I., Dewhirst F.E. (2005). Defining the normal bacterial flora of the oral cavity. J. Clin. Microbiol.

[2] Kolenbrander P.E., Andersen R.N., Blehert D.S., Egland P.G., Foster J.S., Palmer R.J. (2002). Communication among oral bacteria. Microbiol. Mol. Biol. Rev.

[3] Burgess N.A.D., Kirke D.F., Williams P., Winzer K., Hardie K.R., Meyers N.L., Aduse-Opoku L., Curtis M.A., Cámara M. (2002). LuxS-dependent quorum sensing in *Porphyromonas gingivalis* modulates protease and haemagglutinin activities but is not essential for virulence. Microbiology.

[4] Chung W.O., Park Y., Lamont R.J., McNab R., Barbieri B., Demuth D.R. (2001). Signaling system in *Porphyromonas gingivalis* based on a LuxS protein. J. Bacteriol.

[5] Fong K.P., Chung W.O., Lamont R.J., Demuth D.R. (2001). Intra- and interspecies regulation of gene expression by *Actinobacillus actinomycetemcomitans* LuxS. Infect. Immun.

[6] Frias J., Olle E., Alsina M. (2001). Periodontal pathogens produce quorum sensing signal molecules. Infect. Immun.

[7] Merritt J., Qi F., Goodman S.D., Anderson M.H., Shi W. (2003). Mutation of *luxS* affects biofilm formation in *Streptococcus mutans*. Infect. Immun.

[8] Fuqua C., Winans S.C., Greenberg E.P. (1996). Census and consensus in bacterial ecosystems: The LuxR-LuxI family of quorum-sensing transcriptional regulators. Annu. Rev. Microbiol.

[9] Fuqua C., Parsek M.R., Greenberg E.P. (2001). Regulation of gene expression by cell-to-cell communication: Acyl-homoserine lactone quorum sensing. Annu. Rev. Genet.

[10] Williams P., Winzer K., Chan W., Cámara M. (2007). Look who’s talking: Communication and quorum sensing in the bacterial world. Philos. Trans. R. Soc. Lond. B. Biol. Sci.

[11] Schauder S., Bassler B.L. (2001). The languages of bacteria. Genes Dev.

[12] Chan K.G., Puthucheary S.D., Chan X.Y., Yin W.F., Wong C.S., See Too W.S., Chua K.H. (2010). Quorum sensing in *Aeromonas* species isolated from patients in Malaysia. Curr. Microbiol.

[13] Whittaker C.J., Klier C.M., Kolenbrander P.E. (1996). Mechanisms of adhesion by oral bacteria. Annu. Rev. Microbiol.

[14] Winson M.K., Swift S., Fish L., Throup J.P., Jorgensen F., Chhabra S.R., Bycroft B.W., Williams P., Stewart G.A.S.B. (1998). Construction and analysis of *luxCDABE*-based plasmid sensors for investigating *N*-acyl homoserine lactone-mediated quorum sensing. FEMS Microbiol. Lett.

[15] Farrand S.K., Hwang I., Cook D.M. (1996). The *tra* region of the nopaline-type *Ti* plasmid is a chimera with elements related to the transfer systems of RSF1010, RP4, and F. J. Bacteriol.

[16] Chan K.G., Yin W.F., Sam C.K., Koh C.L. (2009). A novel medium for the isolation of *N*-acylhomoserine lactone-degrading bacteria. J. Ind. Microbiol. Biotechnol.

[17] Chan K.G., Atkinson S., Mathee K., Sam C.K., Chhabra S.R., Cámara M., Koh C.L., Williams P. (2011). Characterization of *N*-acylhomoserine lactone-degrading bacteria associated with the *Zingiber officinale* (ginger) rhizosphere: Co-existence of quorum quenching and quorum sensing in *Acinetobacter* and *Burkholderia*. BMC Microbiol.

[18] Chan K.G., Tiew S.Z., Ng C.C. (2007). Rapid isolation method of soil bacilli and screening of their quorum quenching activity. As. Pac. J. Mol. Biol. Biotech.

[19] Chan K.G., Wong C.S., Yin W.F., Sam C.K., Koh C.L. (2010). Rapid degradation of *N*-3-oxo-acylhomoserine lactones by a *Bacillus cereus* isolate from Malaysian rainforest soil. Antonie van Leeuwenhoek.

[20] Lane D.L., Pace B., Olsen G.J., Stahl D.A., Sogin M.L., Pace N.R. (1985). Rapid determination of 16S ribosomal RNA sequences for phylogenetic analyses. Proc. Natl. Acad. Sci. USA.

[21] Tamura K., Dudley J., Nei M., Kumar S. (2007). MEGA4: Molecular Evolutionary Genetics Analysis (MEGA) software version 4.0. Mol. Biol. Evol.

[22] Shaw P.D., Ping G., Daly S.L., Cha C., Cronan J.E., Rinehart K.L., Farrand S.K. (1997). Detecting and characterizing *N*-acyl-homoserine lactone signal molecules by thin-layer chromatography. Proc. Natl. Acad. Sci. USA.

[23] Wong C.S., Yin W.F., Choo Y.M., Sam C.K., Koh C.L., Chan K.G. (2012). Coexistence of quorum-quenching and quorum-sensing in tropical marine *Pseudomonas aeruginosa* strain MW3A. World J. Microbiol. Biotechnol.

[24] Ortori C.A., Atkinson S., Chhabra S.R., Cámara M., Williams P., Barret D.A. (2007). Comprehensive profiling of *N*-acylhomoserine lactones produced by *Yersinia pseudotuberculosis* using liquid chromatography coupled to hybrid quadrupole-linear ion trap mass spectrometry. Anal. Bioanal. Chem.

[25] Wang H., Cai T., Weng M., Zhou J., Cao H., Zhong Z., Zhu J. (2006). Conditional production of acyl-homoserine lactone-type quorum-sensing signals in clinical isolates of enterobacteria. J. Medical. Microbiol.

[26] Yates E.A., Philipp B., Buckley C., Atkinson S., Chhabra S.R., Sockett R.E., Goldner M., Dessaux Y., Cámara M., Smith H., Williams P. (2002). *N*-acylhomoserine lactones undergo lactonolysis in a pH-, temperature-, and acyl chain length-dependent manner during growth of *Yersinia pseudotuberculosis* and *Pseudomonas aeruginosa*. Infect. Immun.

[27] Pritchard D.I. (2006). Immune modulation by *Pseudomonas aeruginosa* quorum-sensing signal molecules. Int. J. Med. Microbiol.

